# Nano wear particles and the periprosthetic microenvironment in aseptic loosening induced osteolysis following joint arthroplasty

**DOI:** 10.3389/fcimb.2023.1275086

**Published:** 2023-10-03

**Authors:** Yu Xie, Yujie Peng, Guangtao Fu, Jiewen Jin, Shuai Wang, Mengyuan Li, Qiujian Zheng, Feng-Juan Lyu, Zhantao Deng, Yuanchen Ma

**Affiliations:** ^1^ Department of Orthopedics, Guangdong Provincial People’s Hospital (Guangdong Academy of Medical Sciences), Southern Medical University, Guangzhou, China; ^2^ Shantou University Medical College, Shantou, China; ^3^ Department of Endocrinology, The First Affiliated Hospital of Sun Yat-sen University, Guangzhou, China; ^4^ The Sixth Affiliated Hospital, School of Medicine, South China University of Technology, Guangzhou, China

**Keywords:** microenvironment, homeostatic imbalance, joint prothesis, joint arthroplasty, aseptic loosening

## Abstract

Joint arthroplasty is an option for end-stage septic arthritis due to joint infection after effective control of infection. However, complications such as osteolysis and aseptic loosening can arise afterwards due to wear and tear caused by high joint activity after surgery, necessitating joint revision. Some studies on tissue pathology after prosthesis implantation have identified various cell populations involved in the process. However, these studies have often overlooked the complexity of the altered periprosthetic microenvironment, especially the role of nano wear particles in the etiology of osteolysis and aseptic loosening. To address this gap, we propose the concept of the “prosthetic microenvironment”. In this perspective, we first summarize the histological changes in the periprosthetic tissue from prosthetic implantation to aseptic loosening, then analyze the cellular components in the periprosthetic microenvironment post prosthetic implantation. We further elucidate the interactions among cells within periprosthetic tissues, and display the impact of wear particles on the disturbed periprosthetic microenvironments. Moreover, we explore the origins of disease states arising from imbalances in the homeostasis of the periprosthetic microenvironment. The aim of this review is to summarize the role of relevant factors in the microenvironment of the periprosthetic tissues, in an attempt to contribute to the development of innovative treatments to manage this common complication of joint replacement surgery.

## 1 Introduction

Septic arthritis occurs when bacteria invade the joint cavity, resulting in the infection of joint cavity and joint function impairment. Joint arthroplasty is an option for end-stage septic arthritis combined with successful control of infection by anti-biotic medications. It is also the surgical option for end-stage hip and knee arthritis due to developmental anomaly, degenerative changes, or autoimmune diseases. This surgical procedure replaces part of the arthritic joint with a plastic, metal, or ceramic prosthesis, aiming to restore normal joint function and improve patients’ quality of life. The evolution of joint replacement techniques has come a long way since the first artificial hip prosthesis was implanted in 1891, using ivory femoral heads (Trebše and Mihelič, 2012), with millions of patients benefiting from this procedure (Katti, 2004; Huang et al., 2012; Singh et al., 2019).

Despite the advances in the biomaterials and improved biocompatibility over the years, complications such as osteolysis persist due to the inevitable release of wear particles (Kapadia et al., 2015; Hodges et al., 2021). The National Joint Registry database records millions of annual primary joint replacement surgeries, with approximately 4-6% requiring joint revision after 10 years, and the revision rate increases over time. Aseptic loosening, characterized by the unexplained loosening of the joint prosthesis without mechanical causes or infection, is the leading cause of joint revision, accounting for over 30% of revision surgeries. Consequently, tens of thousands of individuals undergo revision arthroplasty each year. Understanding the pathogenesis following joint arthroplasty is crucial for the prevention and treatment of these complications (Sadoghi et al., 2013).

Current studies on tissue pathology after prosthesis implantation have identified various cell populations involved in the process, including osteoblasts, osteoclasts, osteocytes, fibroblasts, macrophages, and inflammatory cells. However, there is a paucity of literature describing the interaction between these cells and the altered periprosthetic microenvironment. The periprosthetic microenvironment is a highly intricate environment comprising various cells and extracellular matrix components that work together to maintain microenvironmental homeostasis. Nano wear particles, which is the result of continuous and intense wear of the artificial joint, is a major contributor to the periprosthetic microenvironment and plays an important role in the development of post-implantation inflammation and aseptic loosening.

In this review, we first recall the histological changes occurred after prosthetic implantation, then summarize the cellular changes in the periprosthetic microenvironment, and further put a focus on the disturbance of nano wear particles of the metal implants on the periprosthetic microenvironment. In the last, we also look into cell-cell interactions after prosthetic implantation. Furthermore, we examine previous studies on the mechanisms associated with post-implantation prostheses to gain a better understanding of the changes occurring in the periprosthetic tissue microenvironment and the mechanisms underlying complications. The aim of this review is to summarize the role of relevant factors in the microenvironment of the periprosthetic tissues, and the mechanisms underlying cell-cell and cell-environment interactions caused by imbalances in the periprosthetic tissue microenvironment, therefore contributing to the development of innovative treatments.

## 2 Histological changes after prosthetic implantation

### 2.1 Remodeling of the periprosthetic tissue

**Figure 1:**
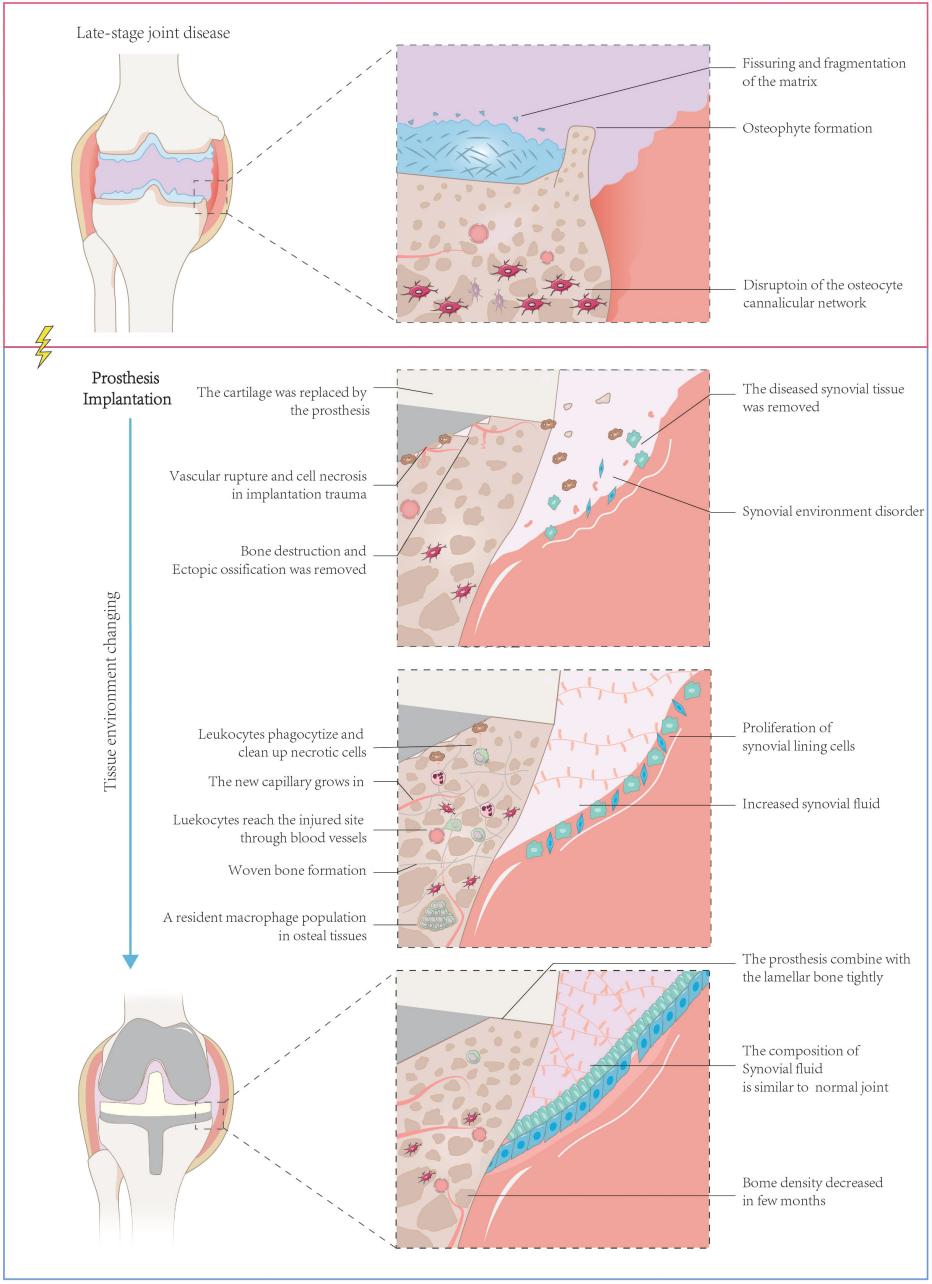
Structure of the artificial joint structure and sequential changes after prosthesis implantation. Artificial joint implantation for end-stage joint disease involves a process of removing damaged cartilage, synovial tissue, and osteophytes, followed by implanting a prosthesis composed of bone and artificial elements. The initial removal leads to joint tissue trauma and potential tissue necrosis, along with diminished synovial function. The body’s reparative processes subsequently get triggered, which involves phagocytosing necrotic tissue, forming woven bone, regrowing microvessels, and repairing synovial tissue, and increasing synovial fluid production. Over time, the prosthesis tightly integrates with the bone, although a decrease in bone density may occur post-implantation. Despite this, synovial function is ultimately restored, and the composition of synovial fluid in the artificial joint mirrors that of a normal one.

The normal joint structure consists of bone, cartilage, and the synovial joint (Wright et al., 1973). After joint arthroplasty, the new composition of the joint includes bone, prosthesis, synovial joint, its newly formed synovial tissue, and joint synovial fluid (Athanasou, 2002) (**Figure 1**). The bone undergoes a series of healing processes, including inflammation, hematoma formation, reparative tissue formation, callus formation, and bone remodeling and maturation (Gallo et al., 2013). Cartilage repair may also occur, but the rate and extent of repair can vary and may not occur in all cases.

In the immediate aftermath of surgery, the body responds to the surgical trauma and the presence of the foreign object (the prosthesis) with an acute inflammatory response. This response involves the accumulation of fluid and immune cells around the prosthesis. Hematoma formation is a natural outcome of this inflammation and plays a vital role in the bone healing process. It occurs when blood leaks from damaged blood vessels and accumulates in the surrounding tissue, providing growth factors and a barrier between the prosthesis and the surrounding tissues. Subsequently, reparative tissue, also known as granulation tissue, forms around the implant. This tissue consists of fibroblasts and blood vessels and acts as a scaffold for new bone formation. Over time, the reparative tissue becomes calcified and forms a callus, which provides support to the surrounding bone. In the final phase of bone healing, the callus undergoes remodeling and maturation. This process allows the bone to regain its original strength and structure while integrating the prosthesis into the surrounding bone. Eventually, the artificial joint replaces the original joint as part of the joint (Athanasou, 2002).

Following the removal of chronically inflamed synovial tissue during joint replacement surgery, over time residual synovial tissue and regenerated synovial tissue form a new synovial component, which contains a lining layer and a sub-lining layer. The synovium is composed of highly vascularized and fibrotic connective tissue infiltrated by macrophages and dendritic cells. The regenerated synovial tissue covers the implant and serves as a smooth gliding surface for joint movement. Periprosthetic pseudomembranes are often observed in pathological states such as aseptic loosening, indicating a poor prognosis for prosthetic implantation (Konttinen et al., 2001; Kung et al., 2015). The combined synovial and periprosthetic membranes are referred to as the “synovial-like interface membrane” (SLIM). The synovial fluid produced by the new synovial tissue contains hyaluronic acid, lubricin, and various phosphatidylcholines, with protein and phospholipid concentration levels comparable to normal synovial fluid. However, the concentration of hyaluronic acid decreases, reducing fluid viscosity and increasing the risk of joint abrasion (Mazzucco et al., 2004).

### 2.2 Appearance of nano wear particles after prosthetic implantation

Elderly patients undergo a significant number of gait cycles per year, ranging from 500,000 to 1 million (Goodman et al., 2014). This continuous and intense wear action exerts strain on the artificial joint, resulting in the generation of wear particles, referred to as prosthetic debris. These wear particles are dispersed throughout the joint fluid along the bone-implant interface (Schmalzried et al., 1992; Revell, 2008). The quantity, size, and origin of these particles influence the extent of bone loss and the number and depth of resorption sites (Saleh et al., 2004; Goodman, 2007). Furthermore, the presence of wear particles in the periprosthetic microenvironment can induce the accumulation of inflammatory cells, leading to bone destruction and disruption of the microenvironment (Jacobs et al., 2006).

### 2.3 The onset of aseptic loosening

When the equilibrium of the prosthetic microenvironment is disrupted by factors such as wear particles, it can give rise to the development of disease states, notably aseptic loosening, where the rate of particle accumulation can surpass the body’s ability to maintain microenvironmental balance, resulting in periprosthetic osteolysis (PPOL) (Shanbhag et al., 1994; Maloney and Smith, 1996; Shanbhag et al., 2002). Aseptic loosening often accompanies periprosthetic osteolysis and an inflammatory response. Additionally, it stands as a significant reason for revision arthroplasty (Maradit Kremers et al., 2015; Singh et al., 2019). Therefore, although significant advancements have been made in prosthetic materials, it remains crucial to prolong the lifespan of prosthetics and minimize particle production to mitigate the risk of aseptic loosening (Couto et al., 2020).

## 3 The cellular periprosthetic microenvironment after prosthetic implantation

The microenvironment refers to the extracellular matrix (ECM) and cells that surround and support target cells. In the context of arthroplasty and prosthesis implantation, the microenvironment encompasses the prosthesis itself, released wear particles, bone-forming lineage cells, immune cells, fibroblasts, and the ECM, which forms a complex network structure that influences the behavior of cells within the microenvironment and plays a crucial role in maintaining homeostasis (Buckley et al., 2021). Here we first analyze the cellular components in the periprosthetic microenvironment, which include immune cells, osteogenic lineage cells forming the joint, and other cells associated with tissue repair processes.

### 3.1 Activation of immune cells in the periprosthetic microenvironment

**Figure 2:**
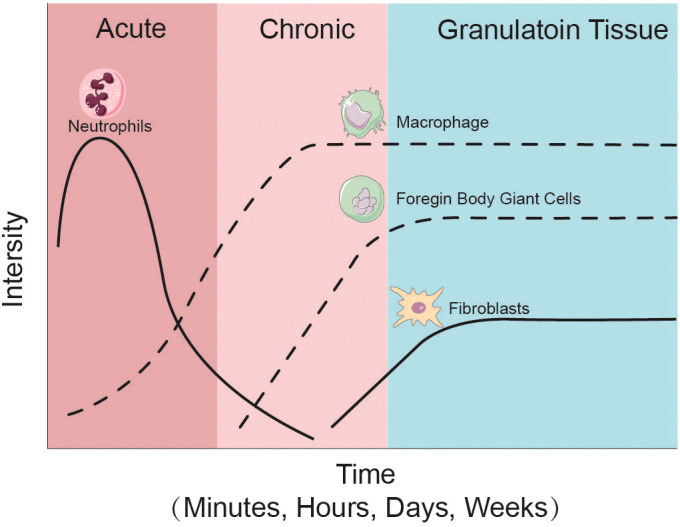
Cellular changes after joint implantation. During the initial phase of prosthesis implantation, neutrophils, as representatives of the acute inflammatory response, are the first to enter the cellular repair response at the site of injury, followed by macrophages and foreign body giant cells as representative cells of the chronic response in the prosthetic microenvironment. At the same time the repair of new capillaries and fibrous tissues gradually proceeds and eventually granulation tissue is formed.

**Figure 3:**
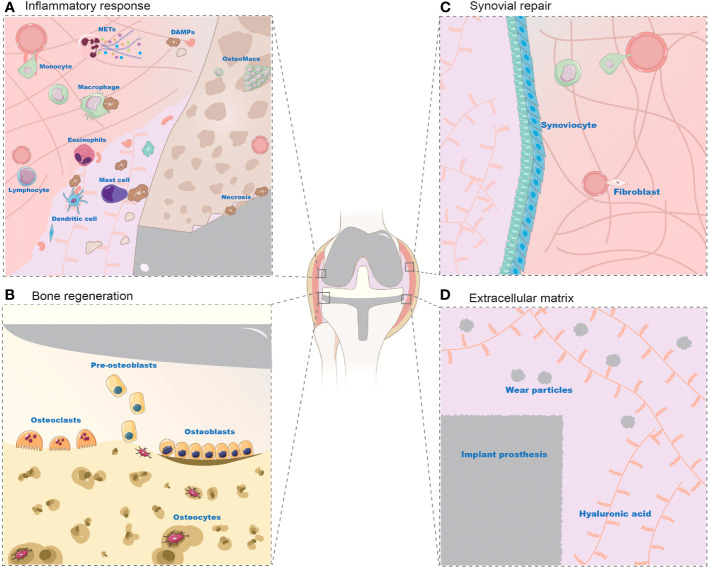
Cell changes in periprosthetic environment after prosthesis implantation. **(A)**: Inflammatory Response Post-Implantation **(B)**: Bone Regeneration Post-Implantation **(C)**: Synovial Repair Post-Implantation **(D)**: Extracellular Matrix Post-Implantation The prosthetic microenvironment comprises cells and extracellular matrix, playing roles in inflammatory response, bone regeneration, and synovial repair. Cells like neutrophils, monocytes, eosinophils, mast cells, dendritic cells, and lymphocytes contribute to the inflammatory response. Neutrophils are the first to converge on the damaged area, eliminating DAMPs (necrotic cells and bone debris) via NETs (extracellular traps). Monocytes migrate to the prosthetic microenvironment, differentiating into macrophages to phagocytose necrotic cells, alongside tissue-resident macrophages. However, the exact mechanisms of eosinophils, mast cells, dendritic cells, and lymphocytes are not fully elucidated. During bone regeneration, mesenchymal stem cells differentiate into osteoblasts to stimulate osteogenesis. Osteoblasts subsequently encapsulated by bone form osteocytes, while osteoclasts are responsible for bone resorption. The synovial membrane consists of synovial-like and fibroblast-like cells, with macrophages aiding in its regeneration and endothelial cells participating in tissue repair. The extracellular matrix, largely studied in the context of wear particles, is an inevitable byproduct in the prosthetic microenvironment, dispersing within the synovial fluid and tissue areas of the joint.

Various types of immune cells respond to prosthetic implantation. Leukocytes, such as neutrophils and monocytes, migrate from the vascularized tissue to the site of damage during the acute inflammatory response, as illustrated in **Figure 2** and **Figure 3A**. As illustrated in **Figure 2**, neutrophils release proteases, lysozymes, and reactive radicals in the form of extracellular traps (NETs). This process contributes to opsonization, clearance, and scavenging at the implant site (Jhunjhunwala et al., 2015; Jorch and Kubes, 2017). However, neutrophils appear transiently and are subsequently replaced by macrophages. Prolonged accumulation of neutrophils after a metal implant may indicate a potential adverse reaction to the metal implant (Grammatopoulos et al., 2016). Mast cells also participate in the acute inflammatory response to the implant by releasing histamine, which recruits macrophages to the implant site by inducing the expression of adhesion molecules on endothelial cells (Zdolsek et al., 2007). Simultaneously, macrophages produce IL-1α, IL-1β, and TGFβ, which recruit aggregates of neutrophils (St. Pierre et al., 2010; Akbar et al., 2012). Macrophages can originate from resident macrophages in bone or differentiate from monocytes in blood vessels. They play a crucial role in clearing the debris at injury site during the subsequent inflammatory response. Eosinophils and dendritic cells are also observed in the acute inflammatory response. Dendritic cells play a role in inducing and programming T cells, while the specific role of eosinophils remains unclear (Keselowsky and Lewis, 2017).

In the adaptive immune response triggered by prostheses, particularly metal implants, lymphocytes, including T cells and B cells, play a crucial role in the normal biological response to the implant (U.S. Food and Drug Administration, 2019). CD4+ T helper cells (Th) exhibit diverse functional responses to different metal components, including proliferation, expansion, and expression of phenotypic markers associated with activation. Tregs cells promote wound healing by inhibiting the aggregation of inflammatory cells and macrophages (Nosbaum et al., 2016; Revell et al., 2016; Markel et al., 2018). CD8+ T cells can be detected in the vicinity of the prosthetic implant, although their involvement in the implant response remains uncertain (Hallab et al., 2012). Histological evidence finds abundant number of T cells in failed implant tissues. However, the presence of T cells near an implant does not necessarily indicate a maladaptive response (Hasegawa et al., 2016; Paukkeri et al., 2016). The response of B cells to implants is not yet fully understood, but their role in prosthetic implantation may involve B cell-mediated type I, II, and III hypersensitivity reactions. Signs of B cell activation have been observed in failed implants (Mittal et al., 2013).

### 3.2 Activation of osteogenic lineage cells after prosthetic implantation

The trauma caused by prosthetic implantation disrupts bone homeostasis and activates MSCs as well as osteogenic lineage cells, including osteocytes and osteoblasts, leading to new bone formation. As illustrated in **Figure 3B**, MSCs are present in the bone marrow stroma, periosteum, and local microvascular walls, which can differentiate into osteoblasts. During the process of bone formation, MSCs are stimulated by cytokines to differentiate into the osteoblast lineage, resulting in the formation of collagen fibers and bone-like tissue composed of mesenchymal cells, pre-osteoblasts, and osteoblasts (Marco et al., 2005; Kuzyk and Schemitsch, 2011). Osteoblasts play a vital role in bone deposition and implant osseointegration. They are primarily responsible for the synthesis of most bone matrix components, regulation of bone mineralization, and provide the foundation for the growth of new bone tissue. Consequently, osteoblasts play a critical role in postoperative implant osseointegration. Osteoclasts are also involved in implant osseointegration and normal bone remodeling. Activation of osteoclasts leads to bone resorption, followed by the activation of osteoblasts and mineralization of new bone tissue (Zhang et al., 2020). Eventually, a new balance is established in bone regeneration and osteolysis. Osteoblasts that are surrounded by the newly generated bone extracellular matrix differentiate into osteocytes, which constitute the majority of cells within the bone and contribute to maintaining bone homeostasis through their involvement in matrix synthesis, regulation of cytokines, and other functions (Pajarinen et al., 2017).

### 3.3 Alterations in other cells during prosthetic implantation

Fibroblasts and endothelial cells play crucial roles in tissue repair following prosthesis implantation (**Figure 3C**). Fibroblasts migrate to the injury site within 2-10 days of implantation. They contribute to tissue repair by producing ECM, especially type I and type III collagen. Simultaneously, proliferating endothelial cells facilitate the formation of new blood vessels, promoting the development of granulation tissue at the injury site. Over time, the granulation tissue gradually diminishes along with fibroblasts, leaving behind a collagenous scar. M2 Macrophages also play a role in this process by secreting growth factors that stimulate fibroblasts and endothelial progenitor cells, as well as guiding ECM remodeling (Krafts, 2010). Additionally, fibroblasts may contribute to the pathological process of bone resorption through the secretion of pro-inflammatory factors (Koreny et al., 2006).

Post implantation, the synovial membrane consists of a thin layer of cells containing macrophage-like synoviocytes and fibroblast-like synoviocytes, and ECM similar to that of a normal joint. The regeneration capacity of synovial tissue are postulated to be originated from local synovial mesenchymal stem cells (MSCs). MSCs is the precursor for mesenchymal lineage (Huang et al., 2011; Lv et al., 2014), which harbor in many tissue sources (Lv et al., 2012) and become the key cell type for tissue regeneration in recent years (Leung et al., 2014; Deng et al., 2020; Qi et al., 2020; Chen et al., 2022). Some literature also suggests that synovial MSCs may manifest as fibroblast-like synoviocytes (Kung et al., 2015; Li et al., 2019; Li et al., 2020).

## 4 The disturbance of nano wear particles on the periprosthetic microenvironment

**Figure 4:**
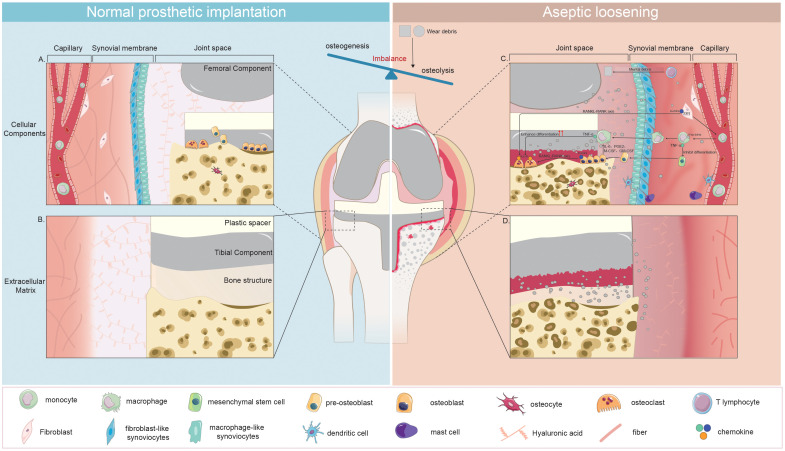
Disruption of Microenvironment Homeostasis in Joint Prosthesis: Cellular and Extracellular Changes Leading to Aseptic Loosening. **(A)**: Cellular Components Post-Implantation with homeostaisis **(B)**: Extracellular Matrix Post-Implantation with homeostaisis **(C)**: Changes in Cellular Components during Aseptic Loosening **(D)**: Changes in Extracellular Matrix during Aseptic Loosening Following implantation, the microenvironment within the joint prosthesis initially attains stability. This is evidenced by the harmonious presence of diverse cell types, including osteoblasts, osteoclasts, osteocytes, synovial cells, and fibroblasts and the establishment of a steady extracellular matrix that includes the prosthesis, bone, and synovium. However, this state of balance is disrupted when aseptic loosening begins. This process initiates as wear particles trigger the differentiation of monocytes into macrophages. The macrophages that form from this differentiation process not only attract additional macrophages but also inhibit the differentiation of osteoblasts by releasing cytokines such as TNF-α. Concurrently, fibroblasts stimulate increased activity in osteoclasts via the RANKL-RANK axis. These changes lead to an overall increase in osteoclast activity and a suppression of osteogenesis. The response also includes participation from mast cells and dendritic cells. In addition, it is suggested that T lymphocytes participate in this process when metal wear particles are generated. The culmination of these alterations results in bone destruction, a roughened surface of the prosthesis, and an increase in synovial inflammation, all of which contribute to aseptic loosening.

A persistent low-grade chronic inflammation could be triggered by nano wear particles surrounding the prosthesis to cause aseptic loosening (Marmotti et al., 2020). As depicted in **Figures 3D**, **Figure 4**, wear particles exhibit resistance to enzymatic degradation and are not readily absorbed by the body, leading to their accumulation in the periprosthetic microenvironment. The persistent chronic foreign body reaction diminishes local bone formation and augments osteolysis, ultimately culminating in aseptic loosening.

### 4.1 Nano wear debris induces inflammation in aseptic loosening

Wear particles primarily activate an innate immune response dominated by macrophages, with the involvement of monocytes, mast cells, and dendritic cells, as illustrated in **Figure 4C**. However, there is still ongoing debate regarding the involvement of lymphocytes in the process of aseptic loosening.

Osteoclasts and tissue-resident macrophages are recognized as the initial cells that encounter wear debris (Lenz et al., 2009; Goodman and Ma, 2010; Smith et al., 2010; Lin et al., 2015). Upon activation, these cells produce pro-inflammatory cytokines and chemokines, which attract monocytes and dendritic cells and amplify the overall inflammatory response. Following the presence of wear particles in the periprosthetic environment, resident macrophages surrounding the prosthesis identify the foreign body through sensing, chemotaxis, phagocytosis, and adaptive stimulation (Medzhitov, 2008). The magnitude of the macrophage response is intensified by larger abrasive particles. When particles are small (<10 µm), individual macrophages and foreign body macrophages can effectively adhere to and phagocytose them. For particles that cannot be efficiently phagocytosed by individual macrophages or foreign body macrophages (20-100 µm), macrophages can fuse together to form multinucleated macrophages or multinucleated foreign body macrophages that surround or isolate large particles, eventually resulting in the formation of foreign body granulomas (Nich et al., 2013). Granulomas comprise histiocytes, fibroblasts, and multinucleated foreign body giant cells (Shen et al., 2006), while monocytes continue to differentiate into macrophages, participating in the reaction during this process.

**Figure 5:**
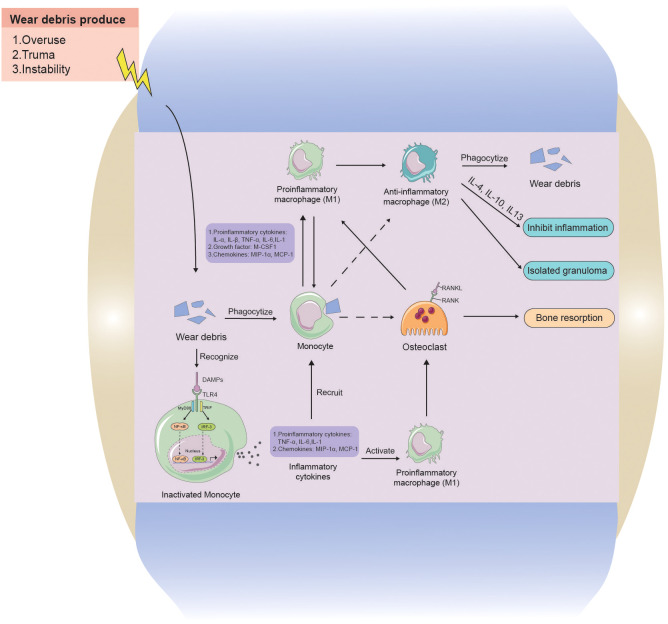
The interaction between wear particles and macrophages in aseptic loosening. Wear particles of artificial joint prosthesis are often released into the prosthetic microenvironment because of overuse, Instability, and trauma factors. Monocytes can swallow wear particles, and when wear particles are swallowed by phagocytes, they will aggravate inflammation by releasing pro-inflammatory cytokines, chemokines, and M-CSF1 to activate M1 phenotype macrophages and promote the release of more inflammatory factors. Whereas osteoclast growth increases, causing increased osteolysis. Monocytes can also recognize stimulatory signals from wear particles through cell contact and release cytokines to further recruit more macrophages, while activating macrophages, causing more osteolysis. M2 phenotype macrophages and M1 phenotype macrophages can interconvert, with M2 phenotype anti-inflammatory macrophages phagocytosing wear particles to lyse and releasing cytokines to inhibit inflammation, as well as encapsulating granulomas to isolate inflammatory lesions.

Current hypotheses propose that wear particle-engulfing macrophages exhibit an MΦ phenotype, and wear particles induce the polarization of macrophages into a pro-inflammatory M1 phenotype. This, in turn, promotes osteoclast maturation, leading to increased bone resorption and periprosthetic osteolysis (Mandelin et al., 2004; Masui et al., 2004; Sabokbar et al., 2015; O’Brien et al., 2016) (**Figure 5**). Simultaneously, macrophages cause further macrophage aggregation through the release of pro-inflammatory cytokines, such as interleukin 1α (IL1-α), IL1-β, tumor necrosis factor α (TNF-α), IL-6, IL-1, growth factors such as macrophage colony-stimulating factor-1), and chemokines such as macrophage inflammatory protein-1 α (MIP-1a) and monocyte chemoattractant protein-1 (MCP-1) (Gibon et al., 2017). Other macrophages, not activated by phagocytosis, undergo polarization through membrane receptor interactions by toll-like receptor 4 (TLR4), CD11b, CD14, wherein TLR4 is induced to activate the nuclear factor kappa-B (NF-κB) pathway primarily via the adapter protein myeloid differentiation primary response gene 88 (MyD88) or directly through the interferon regulatory Factor 3 (IRF3) pathway, resulting in cytokine release. Our previous study revealed a significant downregulation of sirtuin 1 (SIRT1) in macrophages stimulated by metal nanoparticles via the NF-κB pathway (Deng et al., 2017a). Following activation, an increased number of macrophages contribute to an enhanced osteolytic effect (Akira et al., 2001; Tuan et al., 2008). Moreover, macrophages also play a role in fibrosis and attempt tissue repair and restoration. During this phase, M2 phenotype macrophages exhibit an anti-inflammatory function by releasing cytokines (IL-4, IL-10, IL-13), regulating ongoing tissue damage, isolating granuloma-like structures, and attempting to isolate nondegradable materials (Mosser and Edwards, 2008; Purdue, 2008; Sun et al., 2021).

Infiltration of mast cells (Solovieva et al., 1996; Qiu et al., 2005) and dendritic cells (Kou and Babensee, 2010; Vaculová et al., 2018) can be observed in the periprosthetic microenvironment during aseptic loosening (**Figure 4C**). However, the mechanisms behind their involvement remain poorly understood. Concerning the effect of wear particles on dendritic cells (DCs), *in vitro* exposure to ultra-high-molecular-weight polyethylene (UHWMP) particles has demonstrated that wear particles can stimulate major histocompatibility complex (MHC) II expression and IL-12 production by activating TLR1/2 on the surface of DCs (Maitra et al., 2008). Furthermore, the inability of DCs to digest wear particles following phagocytosis leads to lysosome rupture and the release of histone proteases S and proteases B into the cytoplasm, triggering activation of pattern recognition receptors (PRRs) such as the NLR family pyrin domain containing 3 (NLRP3) inflammasome. This activation results in the release of IL-18 and IL-1β from the cells into the extracellular environment. In turn, these cytokines contribute to extracellular matrix lysis, the onset of periprosthetic inflammation, and bone resorption, ultimately leading to the development of osteolysis (Maitra et al., 2009).

Based on current literature, it has been established that lymphocytes play a crucial role in the development of aseptic lymphocyte-dominated vasculitis-associated lesion (ALVAL) in response to wear particle stimulation. However, the role of lymphocytes in aseptic loosening induced by wear particles remains controversial. Some studies have observed a greater aggregation of CD3+ T cells, particularly in metal-on-metal prostheses, in pathological tissue specimens (Hercus and Revell, 2001; Hopf et al., 2017). Conversely, other studies have reported lower levels of T cells in osteolysis tissue and the absence of cytokine release associated with T cells (Li et al., 2001). Furthermore, no significant increase in Th1, Th2, and CD3+ T cells was observed in osteolytic tissue compared to non-osteolytic tissue (Arora et al., 2003; Dapunt et al., 2014). Based on the available literature, it can be presumed that lymphocytes may not play a significant role in aseptic loosening. Existing studies, which are limited by small sample sizes, have primarily reported an increase in T lymphocytes in metal implants, potentially due to the coexistence of ALVAL. Therefore, further investigation is necessary to clarify the role of lymphocytes in aseptic loosening.

### 4.2 Impact of wear particles on bone forming lineage cells

The impact of wear particles on bone-forming lineage cells (MSCs, osteoblasts, osteocytes) is crucial in the osteolysis process within the prosthesis microenvironment in aseptic loosening (**Figure 4C**).

An increasing number of studies have demonstrated that wear particles not only activate macrophages and osteoclasts, leading to increased bone resorption, but also cause significant damage to MSCs. This damage prevents osteoblast differentiation and impairs bone formation, reducing cell viability and impairing the production of mineralized bone matrix (Goodman et al., 2006; O’Neill et al., 2013; Ebert et al., 2021). Animal experiments and cellular studies have revealed that wear particles inhibit MSCs’ osteogenic differentiation, induce the production of pro-inflammatory cytokines such as IL-1β, IL-6, and TNF-α, upregulate receptor activator of nuclear factor-kappa B ligand (RANKL), and decrease osteoprotegerin (OPG) (Cai et al., 2013; Jiang et al., 2013). In response to titanium (Ti) particles, impaired MSCs activity and osteogenic differentiation depend on the phagocytosis of Ti particles. Additionally, granulocyte-macrophage colony stimulating factor (GM-GSF) disrupts cytoskeletal organization and induces MSCs to secrete IL-8 and GM-CSF, further reducing cell viability and osteogenic differentiation similar to the effects of phagocytosis of Ti particles (Okafor et al., 2006; Haleem-Smith et al., 2011). Although several signaling pathways have been observed to have adverse effects on MSCs due to wear particles, such as the NF-κB signaling pathway adversely affecting osteogenic differentiation (Lin et al., 2014), reduced involvement of the Wnt/β-actin signaling pathway in MSC differentiation to osteogenesis (Wang et al., 2016) and activation of the extracellular regulated protein kinases (ERK) signaling pathway (Lee et al., 2011), the number of studies on this topic is limited, and the signaling pathways involved in the inhibition of MSC differentiation induced by wear particles remain poorly understood.

Simultaneously, wear particles stimulate MSCs to express metalloproteinases through multiple signaling pathways. These metalloproteinases can cleave the collagen-rich, mineralized ECM of bone (Chen et al., 2018). Consequently, when matrix metalloproteinases are overexpressed and attach to the surface of the bone-prosthetic tissue, they can degrade the bone matrix and exacerbate periprosthetic osteolysis (Takei et al., 2004; Jonitz-Heincke et al., 2016).

Osteoblasts primarily interact with wear particles through phagocytosis, involving the internalization of the particles (Chiu et al., 2009, 5; Vermes et al., 2016). They also engage in non-phagocytic interactions with the particles (Granchi et al., 2003; Vermes et al., 2006). When particles enter the cytoplasm of osteoblasts, attachment to different cells causes swelling of intracellular organelles and rupture of cell membranes. Furthermore, particles can lead to DNA damage and activate DNA repair mechanisms, although no particles have been observed within the nucleus (Lee et al., 2013; Ribeiro et al., 2016). When particles attach to actin fibers, they significantly disrupt the cytoskeletal structure of osteoblasts, impeding cell function (Saldaña and Vilaboa, 2009; Lee et al., 2011; Lee et al., 2013). Our study has shown that the expression of SIRT1 is significantly downregulated in osteoblasts treated with particles. Additionally, particles can stimulate the production of inflammatory cytokines and induce apoptosis in osteoblasts through the NF-κB pathway (Deng et al., 2017b). Furthermore, our results suggest that the STAT/IL-6 pathway may mediate nanoparticle-induced inflammation and stimulate osteoclast formation (Deng et al., 2021). Moreover, particles inhibit the osteogenic differentiation of osteoblasts through the Wnt/β-catenin and bone morphogenic protein (BMP)/smad signaling pathways (Preedy et al., 2015; Nam et al., 2017; Sun et al., 2019). By secreting extracellular matrix (mainly type I collagen) and matrix metalloproteinases along with their inhibitors such as tissue inhibitor of metalloproteinase (TIMP), osteoblasts wearing particles can inhibit the formation of type I precollagen through the NF-κB signaling (Roebuck et al., 2006; Vermes et al., 2006). This disruption also disturbs the balance between osteogenic matrix metalloproteinases and TIMP (Ma et al., 2006; Syggelos et al., 2013), resulting in decreased osseointegration and subsequent implant failure.

Osteoblasts reside in the mineralized matrix lumen and account for 90-95% of the cells in mineralized bone, with osteoclasts and osteocytes comprising only about 5% of the cells (Shiflett et al., 2019). In response to wear particle stimulation, osteoblasts shift from anabolic to catabolic, as evidenced by increased expression of catabolic markers such as cathepsin K and tartrate-resistant acid phosphatase (TRAP). This leads to periluminal remodeling, causing a significant increase in osteocyte lumen size (Atkins et al., 2009; Ormsby et al., 2016). Interestingly, according to Ormsby’s study, osteoblast-induced bone loss in response to wear particles appears to be gender-specific, affecting only women (Ormsby et al., 2019).

### 4.3 Impact of wear particles on fibroblasts in the synovial membrane

The composition of the synovial membrane in aseptic loosening includes synovial tissue, regenerated synovial tissue, and the periprosthetic membrane between bone and cement or bone and implant, with fibroblasts comprising 70% of these components. During the dispersion of joint fluid along the bone-implant interface, wear particles can persist in the synovial membrane, leading to fibroblasts’ involvement in wear particle-induced osteolysis (Rose et al., 2012). Wear particles can induce the expression of receptor activator of nuclear kappa-B (RANKL) in fibroblasts through various pathways, including the TLR-MyD88-RANKL pathway, the endoplasmic reticulum (ER) stress pathway, and the prostaglandin E2 (PGE2) receptor EP4 signaling pathway, thereby stimulating osteoblast differentiation (Tsutsumi et al., 2009; Wang et al., 2015; Li et al., 2018). However, it is important to note that this response may vary among patients.

## 5 Cell-cell interaction

To gain a better understanding of the complex periprosthetic environment and the interactions among different cell types involved in aseptic loosening, it is crucial to investigate cell communication. The periprosthetic environment encompasses osteogenic lineage cells and various immune cells, such as macrophages, osteoclasts, MSCs, osteoblasts, osteocytes, fibroblasts, mast cells, dendritic cells, and lymphocytes. Among these cell types, osteogenic lineage cells and immune cells play significant roles. Due to their spatial proximity, interactions between these cell types are inevitable, and studying these interactions could provide insights into the mechanisms underlying aseptic loosening (Okamoto et al., 2017). While much remains unknown, exploring cell-to-cell interactions studied in the context of osteolysis and other aspects of orthopedics could serve as a valuable starting point for future research aimed at developing new treatments.

### 5.1 Osteoimmunological interactions

Osteoimmunological interactions are essential for maintaining bone homeostasis and play a significant role in bone pathology, particularly in the process of aseptic loosening, where macrophages and osteoclasts dominate the innate immune response. Immune cells have the ability to influence osteoblastic lineage cells, and conversely, osteoblastic lineage cells may also regulate innate immunity in this process.

The proliferation, function and differentiation of MSCs could be affected by their local microenvironments (Huang et al., 2020). Studies exploring the interaction between macrophages and MSCs in aseptic loosening are still limited. Macrophages contribute to MSCs aggregation by secreting chemokines such as macrophage inflammatory protein 1 (MIP1α) and monocyte chemotactic protein-1 (MCP-1) (Huang et al., 2010). Furthermore, macrophages can inhibit the osteogenic differentiation of MSCs. In conditioned medium with Ti particles mimicking macrophage activation, macrophages induce the MSCs-mediated NF-κB signaling pathway in sclerostin via TNF-α. This inhibits Wnt and BMP signaling pathways, resulting in decreased runt-related Transcription Factor 2 (RUNX2) expression, alkaline phosphatase (ALP) activity, and bone mineralization in MSCs (Lee et al., 2012). On the contrary, particle-stimulated macrophages have been shown to stimulate MSCs to promote osteogenesis through IL-10 (Mahon et al., 2020). Recent studies have demonstrated that co-culturing MSCs with M2 macrophages promotes bone formation (Gao et al., 2021). Additionally, it has been observed that M1 macrophages inhibit the growth of MSCs, while M2 macrophages promote their growth (Lu et al., 2021). Therefore, it is possible that M1 macrophages inhibit osteogenic differentiation, whereas M2 macrophages promote bone formation in aseptic loosening.

Regarding the effect of MSCs on immune cells, it has been discovered that MSCs have an inhibitory effect on the inflammatory response. Blocking the secretion of the chemokine C-C-motif receptor (CCR1) in MSCs can lead to an increase in particles-induced osteolysis, suggesting that recruiting MSCs to inflamed areas helps limit inflammation and may contribute to bone regeneration (Gibon et al., 2012). Recent studies have also shown that MSCs can increase the ratio of M2/M1 cells, reducing bone resorption and enhancing bone formation (Shen et al., 2022; Kushioka et al., 2023). While MSCs primarily regulate adaptive immune responses in inflammatory diseases (Bernardo and Fibbe, 2013), an increasing number of studies indicate their crucial role in modulating innate immunity (Le Blanc and Mougiakakos, 2012). MSCs can induce macrophage polarization to an M2 anti-inflammatory phenotype through the paracrine secretion of PGE2, transforming growth factor β (TGF-β), indoleamine 2,3-dioxygenase (IDO), chemokine C-C motif ligand 2 (CCL2), and chemokine C-X-C Motif ligand 12 (CXCL12) (Pajarinen et al., 2017; Lu et al., 2021). Thus, MSCs may suppress the inflammatory response by modulating the innate immune response in aseptic loosening.

Macrophages can aggregate in osteal tissues to form osteomas and are believed to play a crucial role in directing osteoblast function and mineralization. However, the interaction of site-specific macrophage populations with other cells has not been studied yet (Cho, 2015; Miron and Bosshardt, 2015). In the context of aseptic loosening, macrophages inhibit osteoblasts, leading to increased osteolysis. The release of cytokines, such as TNF-α, IL-6, IL-1β, and GM-CSF, stimulates the secretion of osteoblasts, including IL-6, PGE2, M-CSF, GM-CSF, MCP-1, and RANKL (Rodrigo et al., 2002; Vallés et al., 2008; Guo et al., 2013), which further recruit macrophages, increase osteoclast production, and inhibit osteoblast function. Osteoblasts can modulate the degree of inflammation in macrophages and regulate the macrophage response to particle stimulation through the release of soluble mediators. In an osteoblast-macrophage co-culture model, lower levels of TNF-α and IL-1β were detected, possibly due to the paracrine action of PGE2 from osteoblasts (Rodrigo et al., 2005). Furthermore, it has been found that when macrophages were co-cultured with osteoblasts, macrophages could produce lipoxin A4 (LXA4) to counteract polymethylmethacrylate (PMMA) induced cytokine production, and this production of LXA4 was only observed in the presence of osteoblasts (Li et al., 2009). These findings suggest that osteoblast-macrophage interactions contribute to the resolution of particle-induced inflammation.

Osteoclasts are the only cells in the body responsible for bone resorption and work together with osteoblasts to maintain the dynamic balance of bone metabolism. Osteoblasts interact with osteoclasts through secreted factors RANKL and osteoprotegerin (OPG). The binding of RANKL and RANK activates the NF-κB pathway, ultimately leading to osteoclast formation, while OPG serves as a decoy receptor for RANKL and negatively regulates osteogenesis. The ratio of RANKL/OPG determines the degree of osteoclast differentiation and function. Wear particles induce a shift from an anabolic phenotype to a catabolic phenotype in osteoblasts (Atkins et al., 2009), resulting in increased expression of cytokines mediating osteoclastogenesis (TNF-α, IL-1β, IL-6, IL-8, PGE2, M-CSF, MCP1, RANKL) and decreased OPG expression (Granchi et al., 2003; Pioletti and Kottelat, 2004; Atkins et al., 2009; Gordon, 2016). Recent studies have also identified a reverse signaling pathway of RANKL in osteoclast-osteoblast coupling, where osteoclasts promote osteoblast bone formation by secreting vesicular RANK and stimulating osteoblast differentiation. Signaling proteins and neurotrophins also play a significant role in the communication between osteoclasts and osteoblasts. Moreover, non-resorbing osteoclasts have been found to influence the function of osteoblasts under particle attack. Interestingly, recent research suggests that the intricate balance of bone homeostasis is maintained through communication between osteoblasts and osteoclasts using exosomes (Yuan et al., 2018). However, the roles of these interactions in aseptic loosening have not been studied extensively, and further research is needed to determine their importance.

The interaction between osteoclasts and MSCs is not well understood, but current research suggests that MSCs can regulate osteoclast activation and formation through paracrine secretion and the RANKL-RANK-OPG pathway. New studies have also shown that MSCs can inhibit osteoclasts, and stem cell exosomes can block osteoclast activation or differentiate into osteoblasts to regulate bone remodeling. BMSC derived exosomes have been demonstrated to activate osteogenesis and downregulate osteoclastogenesis through multiple pathways (Ma et al., 2022).

### 5.2 The interaction of fibroblasts with other cells

Apart from the interaction between immune cells and bone cells, limited research has been conducted on other types of cell-to-cell interactions within implant microenvironments. However, some evidence suggests that fibroblasts, when interacting with osteoclasts, also play a crucial role in maintaining microenvironment homeostasis in aseptic loosening. Studies have shown that fibroblasts in the periprosthetic environment can induce the differentiation of MSCs and peripheral blood mononuclear cells into mature osteoclasts, leading to local bone resorption or osteolysis through mechanisms involving RANKL and TNF-α (Sakai et al., 2002; Sabokbar et al., 2005). Recent research has found that fibroblasts can enhance osteoclast differentiation through X-box binding protein 1 (XBP1) mediated RANKL expression (Wang et al., 2017). Furthermore, fibroblasts on the bone surface can actively participate in bone resorption by degrading the bone matrix through the release of acidic components and bone-degrading enzymes, without the involvement of osteoclasts (Pap et al., 2003). In summary, fibroblasts can accelerate bone resorption around implants by promoting osteoclast differentiation and directly releasing bone-resorbing substances.

The role of osteoclasts in relation to fibroblasts has not been explored in the literature, and there is currently no available research on the interaction between osteogenic lineage cells and fibroblasts. The lack of literature on the interaction between osteoclasts and fibroblasts, as well as between osteoblasts and fibroblasts, highlights the need for further research in this area. Understanding the mechanisms underlying the interaction between these cell types may provide insight into the development and progression of aseptic loosening.

## 6 Conclusion

Aseptic loosening is a complex process involving biomaterials, host tissues, and the immune system. It is characterized by immune cell infiltration, cytokine production, osteoclast activation, bone resorption, and wear debris production. Factors such as long-term wear of the implant and disruption of microenvironment homeostasis contribute to aseptic loosening. Potential therapeutic targets include anti-inflammatory drugs, osteoclast inhibitors, and antioxidants, addressing chronic inflammation, bone resorption, and oxidative stress, respectively.

Despite the progress made in understanding aseptic loosening, current research mainly focuses on wear particle-induced cellular effects, while cell interactions and spatial distribution in the prosthesis remain understudied. Therefore, future studies should concentrate on elucidating cell interactions using advanced technologies such as single-cell and spatial sequencing to uncover the complex relationships between various cell types and their functions in the microenvironment. Moreover, interdisciplinary collaboration among material scientists, biologists, and clinicians is essential for developing innovative solutions that bridge the gap between fundamental research and clinical applications.

Further research should investigate cellular interactions, spatial distribution in the microenvironment, and potential therapeutic targets, with the aim of developing a comprehensive understanding of the mechanisms underlying aseptic loosening. This knowledge will contribute to the development of innovative treatments that effectively prevent and manage this common complication of joint replacement surgery, ultimately improving patient outcomes and quality of life.

## Author contributions

XY: Data curation, Formal Analysis, Investigation, Visualization, Writing – original draft. YP: Data curation, Investigation, Validation, Writing – review & editing. GF: Investigation, Validation, Writing – review & editing. JJ: Investigation, Writing – review & editing. SW: Investigation, Writing – review & editing. ML: Investigation, Writing – review & editing. QZ: Writing – review & editing, Funding acquisition, Supervision. FL: Funding acquisition, Writing – review & editing, Validation. ZD: Conceptualization, Funding acquisition, Project administration, Supervision, Writing – review & editing. YM: Conceptualization, Funding acquisition, Project administration, Supervision, Writing – review & editing.
